# Renal Abscess Secondary to Urinary Tract Obstruction Caused by Pelvic Organ Prolapse: A Report of Two Cases

**DOI:** 10.1002/iju5.70284

**Published:** 2026-10-15

**Authors:** Hiroki Sai, Kumiko Kato, Atsushi Kiyota, Yukiko Tsunoda, Masashi Kato

**Affiliations:** ^1^ Department of Urology Japanese Red Cross Aichi Medical Center Nagoya Daiichi Hospital Nagoya Aichi Japan; ^2^ Department of Urology Nagoya University Graduate School of Medicine Nagoya Aichi Japan; ^3^ Department of Female Urology Japanese Red Cross Aichi Medical Center Nagoya Daiichi Hospital Nagoya Aichi Japan; ^4^ Department of Female Urology Meitetsu Hospital Nagoya Aichi Japan; ^5^ Department of Endocrinology Japanese Red Cross Aichi Medical Center Nagoya Daiichi Hospital Nagoya Aichi Japan

**Keywords:** diabetes mellitus, hydronephrosis, pelvic organ prolapse, pyelonephritis, renal abscess

## Abstract

**Introduction:**

Pelvic organ prolapse can cause urinary tract obstruction, leading to hydronephrosis, pyelonephritis, and in rare cases, renal abscess.

**Case Presentation:**

We report two elderly women with advanced pelvic organ prolapse who developed renal abscesses. Both presented with pyelonephritis and acute renal impairment accompanied by bilateral hydronephrosis. Imaging demonstrated poorly enhanced renal lesions consistent with renal abscess. Infection was successfully controlled with antimicrobial therapy, followed by surgical correction. Renal function improved, and no recurrence was observed during follow‐up.

**Conclusion:**

Advanced pelvic organ prolapse can cause severe urinary tract obstruction, leading to pyelonephritis and, in rare cases, renal abscess. Early recognition of advanced pelvic organ prolapse and urinary dysfunction, prompt infection control, and timely surgical management are essential to prevent serious complications.

## Keynote Message

POP can cause severe urinary tract obstruction, leading not only to hydronephrosis but also to life‐threatening infections. This report highlights how advanced prolapse may progress to renal abscess if urinary dysfunction and infection are not promptly recognized. Early diagnosis, appropriate infection control, and timely surgical correction are essential to prevent serious renal complications.

**Abbreviations**
CT: computed tomography
POP: pelvic organ prolapse
PVR: post‐void residual
SGLT2 inhibitor: sodium–glucose cotransporter 2 inhibitor
TVM: transvaginal mesh
UTI: urinary tract infection

## 1 Introduction

Although hydronephrosis and postrenal renal failure associated with POP have been well documented [1, 2, 3, 4], reports of pyelonephritis remain limited, despite UTI being a relatively common clinical complication of advanced POP [5, 6, 7, 8]. Based on an extensive search of PubMed, renal abscess associated with POP has not previously been reported. Here, we report two cases of renal abscess triggered by urinary dysfunction secondary to POP.

## 2 Case Presentations

### 2.1 Case 1

A 75‐year‐old woman with hypertension, dyslipidemia, rheumatoid arthritis, and hypothyroidism was admitted to our hospital. She had one previous vaginal delivery and had been aware of POP for two years. One month before admission, she began manually reducing the prolapsed bladder to facilitate voiding because of progressive voiding difficulty. She was transported by ambulance after becoming febrile and unable to move at home.

Laboratory tests showed elevated inflammatory markers (C‐reactive protein, 14.5 mg/dL; white blood cell count, 29 400/μL) and severe renal dysfunction (serum creatinine, 11.2 mg/dL). Ultrasonography demonstrated bilateral hydronephrosis. After partial renal recovery, contrast‐enhanced CT revealed multiple poorly enhanced areas in both kidneys, consistent with renal abscesses (Figure 1a,b). Pelvic examination confirmed POP quantification stage IV (Aa +2, Ba +9, C + 9, Ap +0.5, Bp +5.5) (Figure 1c).

**FIGURE 1:**
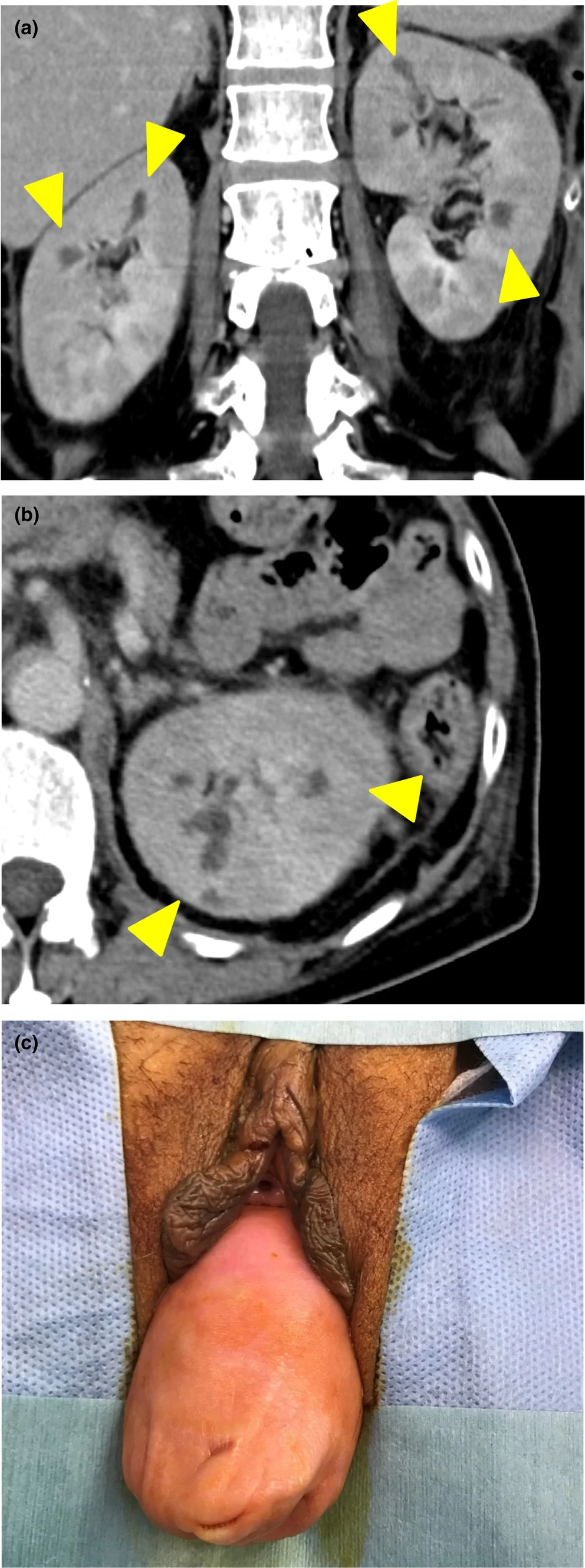
Imaging and intraoperative findings of Case 1. (a, b) Contrast‐enhanced CT shows multiple poorly enhanced areas in both kidneys, consistent with renal abscess formation. Bilateral hydronephrosis had resolved following urethral catheter placement. (a) Coronal view. (b) Axial view. (c) Intraoperative view of the prolapsed pelvic organ in the lithotomy position.

A Foley catheter was initially placed for urinary drainage. The prolapsed pelvic organs were manually reduced and maintained in the reduced position. After two weeks of ceftriaxone and levofloxacin, the infection was controlled, and the catheter was removed. She continued spontaneous voiding with PVR < 50 mL, and TVM surgery and perineorrhaphy were performed in the third week. She did not require self‐catheterization. No pessary was used during the waiting period.

Three months postoperatively, CT revealed improved hydronephrosis, and prolapse had improved to stage I (Aa −2, Ba −2, C −6, Ap −3, Bp −3). No recurrence of POP or infection occurred during two years of follow‐up.

### 2.2 Case 2

A 79‐year‐old woman with poorly controlled diabetes mellitus (hemoglobin A1c, 9.0%; fasting blood glucose level, 373 mg/dL) who was receiving an SGLT2 inhibitor was admitted to our hospital. She had two previous vaginal deliveries. Two months before admission, she had been diagnosed with POP (POP quantification stage IV: Aa +2.5, Ba +4.5, C +4.5, Ap +1.5, Bp +1.5). While awaiting surgery, she developed anorexia and a high fever and was hospitalized.

Laboratory tests showed elevated inflammatory markers (C‐reactive protein, 39.5 mg/dL; white blood cell count, 30 800/μL) and severe renal dysfunction (serum creatinine, 10.5 mg/dL). CT demonstrated bilateral hydronephrosis and a poorly enhanced lesion in the left kidney, suggestive of a renal abscess (Figure 2).

**FIGURE 2:**
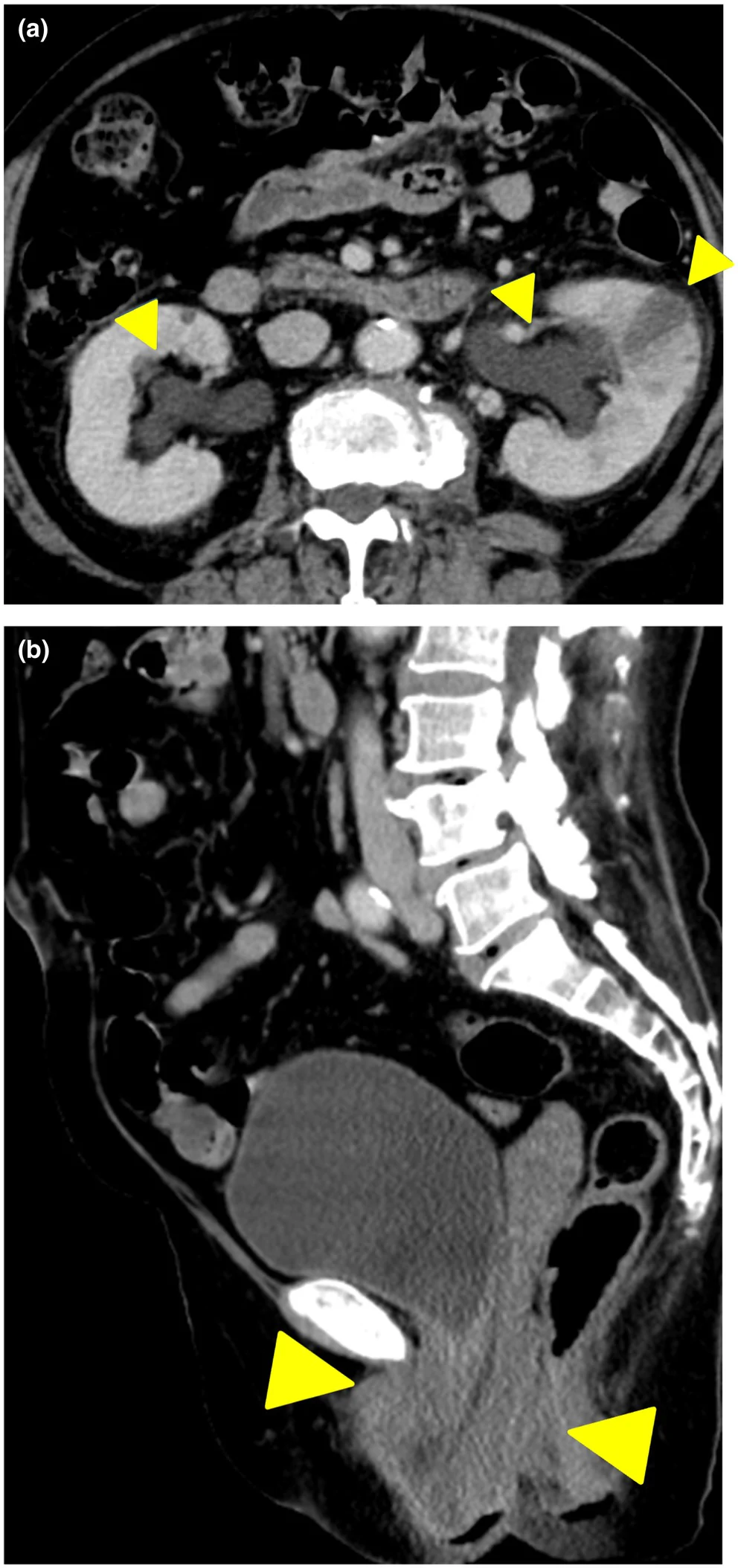
Imaging findings of Case 2. (a) Contrast‐enhanced CT shows bilateral hydronephrosis and a poorly enhanced area in the left kidney, consistent with renal abscess formation. (b) Sagittal view demonstrates the prolapsed pelvic organ.

The SGLT2 inhibitor was discontinued and replaced with a dipeptidyl peptidase‐4 inhibitor, with temporary insulin therapy for 14 days. A Foley catheter was initially placed for urinary drainage. The prolapsed pelvic organs were manually reduced. After two weeks of meropenem and glycemic control, the infection was resolved, and the catheter was removed. She voided spontaneously with PVR < 30 mL until TVM surgery and perineorrhaphy were performed in the third week. She did not require self‐catheterization. No pessary was used.

Three months after surgery, CT revealed improved hydronephrosis, and prolapse had improved to stage I (Aa −3, Ba −3, C −6.5, Ap −3, Bp −3). No recurrence of POP or infection occurred during two years of follow‐up.

## 3 Discussion

POP can cause urinary dysfunction through urethral kinking and increased PVR, predisposing patients to UTI [5, 6, 7, 8, 9]. In advanced POP, bladder and ureteral prolapse toward the urogenital diaphragm may cause bilateral ureteral kinking, while direct uterine compression may further impair drainage [6, 10, 11, 12, 13]. Hydronephrosis occurs in approximately 3.5%–30.6% of patients with POP [1, 2, 3, 4]. Prolonged obstruction may therefore cause hydroureteronephrosis, renal dysfunction, and upper UTI. In our cases, these mechanisms likely contributed to the progression from urinary dysfunction and hydronephrosis to pyelonephritis and renal abscess.

Renal abscess may develop through ascending UTI or hematogenous spread [14]. In patients with advanced POP, persistent obstruction and urinary stasis may facilitate ascending infection and impair resolution of upper UTI. These factors potentially allow the infection to extend into the renal parenchyma. Thus, renal abscess may represent a consequence of prolonged urinary tract obstruction in advanced POP. Although pyonephrosis involves purulent material within an obstructed collecting system [15, 16], the renal parenchymal lesions in our patients were considered renal abscesses based on contrast‐enhanced CT.

Temporary urinary catheterization may be required for severe urinary dysfunction. However, prolonged catheterization may cause urethral or bladder neck erosion [17, 18]. Urethrovaginal fistula has also been reported as a complication associated with catheterization in advanced POP [19]. In both cases, the prolapsed organs were manually reduced and maintained in the reduced position during catheterization. Once adequate spontaneous voiding was confirmed, both patients continued to void spontaneously until surgery. Hydronephrosis improved after surgery with no recurrence during two years of follow‐up.

A pessary was not used in either case because of concerns regarding vaginal mucosal erosion and granulation tissue formation, which could potentially increase the risk of local infection and adversely affect the subsequent surgical procedure. Surgical treatment of POP should be individualized according to the patient's age, general condition, sexual activity, type and severity of prolapse, patient preference, and the surgeon's experience. Laparoscopic or robot‐assisted sacrocolpopexy may be considered when preservation of vaginal function is important, whereas TVM surgery or obliterative procedures such as colpocleisis may be appropriate for selected elderly or frail patients who do not desire future vaginal intercourse. In our two cases, both patients were elderly and had comorbidities, and TVM was selected after discussion of available surgical options.

Advanced age, multiparity, vaginal delivery, and chronic increases in intra‐abdominal pressure are associated with advanced POP [12, 13]. Delayed recognition and treatment may allow prolapse and urinary dysfunction to progress to upper urinary tract complications. In Case 1, limited mobility and insufficient self‐care may have delayed management. In Case 2, poorly controlled diabetes mellitus likely increased susceptibility to severe infection and may have contributed to renal abscess formation [20].

These cases highlight the importance of early recognition of advanced POP and associated urinary dysfunction. Routine gynecological assessment, evaluation of voiding difficulty, and assessment for hydronephrosis may help identify obstruction before severe infection or renal dysfunction develops. When severe infection occurs, prompt antimicrobial treatment and stabilization should be followed by definitive prolapse correction. Early diagnosis and timely intervention may therefore prevent the progression of POP to hydroureteronephrosis, renal dysfunction, severe upper UTI, and potentially, renal abscess.

## 4 Conclusion

We report two cases of renal abscess associated with urinary tract obstruction caused by POP. Advanced POP can cause prolonged obstruction, leading to hydroureteronephrosis, renal dysfunction, and upper UTI, which may rarely progress to renal abscess. Early recognition of advanced POP and urinary dysfunction, prompt infection control, and timely definitive surgical correction are crucial to prevent severe urinary tract complications and recurrence.

## Ethics Statement

The authors have nothing to report.

## Consent

Written informed consent was obtained from both patients for publication of this case report and accompanying images.

## Conflicts of Interest

The authors declare no conflicts of interest.

## Data Availability

The data that support the findings of this study are available from the corresponding author upon reasonable request.
